# Quantitative Platelet Abnormalities in Patients With Hepatitis B Virus-Related Liver Disease

**DOI:** 10.4021/gr2009.12.1329

**Published:** 2009-11-20

**Authors:** Sylvester Chuks Nwokediuko, Obike Ibegbulam

**Affiliations:** a Departments of Medicine, University of Nigeria Teaching Hospital, Ituku/Ozalla, PMB 01129 Enugu, Nigeria; b Haematology, University of Nigeria Teaching Hospital, Ituku/Ozalla, PMB 01129 Enugu, Nigeria

**Keywords:** Platelet, Hepatitis B virus, Liver disease, Paraneoplastic syndrome

## Abstract

**Background:**

Platelets play a central role in primary hemostasis. Quantitative abnormalities of platelets are known to occur in chronic liver disease. The study was carried out to determine the abnormalities of platelet count in various forms of Hepatitis B virus-related liver disease.

**Methods:**

Platelet count was carried out on consecutive chronic liver disease patients seen at the gastroenterology unit of the University of Nigeria Teaching Hospital Ituku/Ozalla who tested positive for Hepatitis B surface antigen (HBsAg) from January 2007 to June 2009. Dyspeptic patients undergoing upper gastrointestinal endoscopy who were HBsAg negative were used as controls.

**Results:**

There were 142 patients with various forms of HBV-related liver disease (asymptomatic infection 29.6%, chronic hepatitis 8.4%, cirrhosis 27.5%, and hepatocellular carcinoma 34.5%). There was no statistically significant difference between the mean platelet count in the patients with Hepatitis B virus (HBV) related liver disease as a whole and control subjects (p = 0.4655). However patients with cirrhosis had a statistically significant lower platelet count than control subjects (p < 0.0001). Conversely, patients with hepatocellular carcinoma (HCC) had a higher platelet count than control subjects (p < 0.0001), and cirrhotic patients (p < 0.0001).

**Conclusions:**

Abnormalities of platelet count occur in HBV-related liver disease. Patients with liver cirrhosis tend to have lower platelet count while patients with HCC tend to have higher counts. Thrombocytosis may be a paraneoplastic manifestation of HCC.

## Introduction

Platelets are the smallest cellular components of human blood, ranging in size from 2 - 4 microns. They are cytoplasmic fragments of the megakaryocyte. Platelets adhere to the site of injury and aggregate with one another, a process known as primary haemostasis.

Platelet disorders can be quantitative or qualitative. Quantitative defects are abnormalities in platelet number, whereas qualitative defects are abnormalities in platelet function. Changes in platelet count accompany the progression of various forms of liver disease including those caused by Hepatitis B virus (HBV) [1]. This explains the use of platelet count as an indirect marker in some of the noninvasive assessments of hepatic fibrosis [2]. Thrombocytopaenia is a common feature of chronic liver disease and has been reported in 49-64% of cirrhotic patient [3]. Conversely, an increased platelet count has been demonstrated in several malignancies, and may be an adverse prognostic indicator in that cancers [4-11]. Hepatocellular carcinoma (HCC) happens to be a malignant disease that is HBV-related.

Approximately 350 million people worldwide have chronic HBV infection [12, 13] and most of them live in South-East Asia and Sub-Saharan Africa [14]. The natural course of HBV chronic infection is variable, ranging from an inactive HBsAg carrier state to a more or less progressive chronic hepatitis, potentially evolving to cirrhosis and HCC [15-17].

Determination of platelet count is a relatively simple laboratory procedure. Close monitoring of platelet count may be a useful tool in the follow-up of patients with chronic HBV infection especially in situations where more complex tests and liver biopsy are not readily available. This study was undertaken to determine if there are quantitative platelet abnormalities in Nigerian patients at various stages of liver disease related to HBV, including HCC which represents the end of the disease spectrum.

## Materials and Methods

This case-control, prospective and cross sectional study was carried out at the gastroenterology unit of the department of medicine, University of Nigeria Teaching Hospital (UNTH) Ituku/Ozalla between January 2007 and June 2009. Consecutive patients with clinical features of chronic liver disease who tested positive for HBsAg constituted the cases. Asymptomatic individuals referred to the unit after testing positive for HBsAg during screening for blood donation or routine medical examination were also included as cases. The study was approved by the UNTH research ethics committee and informed consent was obtained from all the participants. Each participant was first evaluated with detailed history and complete physical examination with emphasis on the hepatobiliary system.

The presence of ascites was specifically documented. Hepatic encephalopathy was graded using the classification adopted at the 11th world congresses of gastroenterology in Vienna [18].

Exclusion criteria: 1, Clinical evidence of infection; 2, Bleeding; 3, Haemolytic disorders; 3, Sickle cell disease; 4, Bone marrow failure; 5, Myeloproliferative disorders.

Hepatitis B surface antigen (HBsAg) was tested for in venous blood using an enzyme-linked immunosorbent assay (ELISA) kit that uses polystyrene microwell strips precoated with monoclonal antibodies specific for HBsAg. Those who tested positive were further evaluated with the following routine laboratory tests for the assessment of patients with chronic liver disease: serum bilirubin, liver enzymes (transaminases and alkaline phosphatase), serum protein (total and albumin), prothrombin time, full blood count including platelet count, urinalysis, abdominal ultrasonography, and where feasible CT scan, HBV DNA and liver biopsy. Platelet count was carried out within 6 hours of sample collection using an automated haematology analyzer (Sysmex XT2000i) manufactured by Sysmex corporation JAPAN.

The control group consisted of patients referred for upper gastrointestinal endoscopy for dyspepsia who did not have alarm symptoms [19], had no clinical evidence of infection or haemorrhage, negative for HBsAg and had no haematological disease. Platelet count was the only additional laboratory test done on the suitable control subjects. All platelet counts were expressed as ×10^9^ /L and for the purpose of this study the normal range was 150 – 400 ×10^9^/L.

Based on the clinical features and results of laboratory tests, the patients were grouped into asymptomatic HBV infection, chronic hepatitis, liver cirrhosis and HCC. Liver disease severity was assessed in the patients with chronic hepatitis, liver cirrhosis and HCC using the Child-Turcotte-Pugh scoring system with the parameters of encephalopathy grade, ascites, prothrombin time, serum albumin and serum bilirubin [20, 21].

### Statistical analysis

The results were analysed with the computer program SPSS version 14 and expressed as means and proportions. Differences between means and proportions were determined and a p value of less than 0.05 was considered significant. Strength of association was determined where indicated using correlation coefficient.

## Results

One hundred and ninety one patients with clinical and laboratory features of chronic liver disease were screened for HBsAg and out of these 142 were positive (74.3%). These consisted of 42 patients with asymptomatic infection (29.6%), 12 patients with chronic hepatitis (8.4%), 39 with cirrhosis (27.5%), and 49 with HCC (34.5%). One hundred and six of these patients (81.7%) were males and 26 (18.3%) were females (Table 1).

**Table 1:** Gender Distribution of HBV-related Liver Disease

Liver Disease	Male	Female	Total (%)
Asymptomatic	36	6	42 (29.6)
Chronic Hepatitis	9	3	12 (8.4)
Cirrhosis	35	4	39 (27.5)
HCC	36	13	49 (34.5)

HCC: Hepatocellular carcinoma

The mean age of the patients with HBV-related liver disease was 44.394 ± 15.154 years while the mean age of the control subjects was 50.638 ± 12.308 years. The difference between the 2 means was not statistically significant (p = 0.9502). The mean platelet count in the HBV-related liver disease patients as a whole was 208.866 ± 128.504 while the mean platelet count in the control subjects was 194.617 ± 62.278. The difference between the means was not statistically significant (p = 0.4655). The mean platelet count in patients with asymptomatic infection was 197.0 ± 66.464. The difference between this mean and the mean platelet count in control subjects was not statistically significant (p = 0.8618). The mean platelet count in patients with chronic hepatitis, cirrhosis and HCC were 200.333 ± 50.879, 105.692 ± 23.933 and 303.245 ± 160.639 respectively. Three patients with HCC (6.1%) had thrombocytosis (platelet count above 400×10^9^/L), Table 2.

**Table 2:** Platelet Count in HBV-related Liver Disease

Liver Disease	Mean Platelet Count (×10^9^/L)
HBV-related liver disease patients (n = 142)	208.866 ± 128.504
Asymptomatic (n = 42)	197.0 ± 66.464
Chronic Hepatitis (n = 12)	200.333 ± 50.878
Cirrhosis (n = 39)	105.692 ± 23.933
HCC (n = 49)	303.245 ± 160.639

HCC: Hepatocellular carcinoma

Table 3 shows comparison of mean platelet counts in the different subgroups of HBV-related liver disease. A test of correlation was carried out between platelet count and Child-Pugh’s score in the patients with cirrhosis. The correlation coefficient (γ) was -0.6183, p= 0.0001. Conversely, a positive correlation was demonstrated between platelet count and Child-Pugh’s score in the patients with HCC (γ = 0.8315, p < 0.0001).

**Table 3:** Comparing Platelet Count At Different Stages of HBV-related Liver Disease

Liver Disease	t value	df	p value
Control versus HBV-related liver disease	0.7313	187	0.4655
Control versus Asymptomatic	0.1746	87	0.8618
Asymptomatic versus Chronic Hepatitis	0.1604	52	0.8732
Asymptomatic versus Cirrhosis	8.102	79	< 0.0001*
Cirrhosis versus HCC	7.604	86	< 0.0001*
Asymptomatic versus HCC	4.0	89	0.0001*
Control versus HCC	4.333	94	< 0.0001*

HCC: Hepatocellular carcinoma. * statistically significant

## Discussion

Hepatitis B virus remains a major aetiologic agent in chronic liver disease in Nigeria as amply demonstrated in this study in which it accounted for 74.3% of all cases. This is similar to an earlier study in the same centre [22].

The mean platelet count in the control subjects was not significantly different from the mean platelet count in the patients with HBV- related liver disease as a whole (p = 0.4655). This is because some of the patients (especially those with cirrhosis) had predominantly thrombocytopaenia while others (especially those with HCC) tended to have higher platelet count. The result was that the mean was somewhere in between. However a comparison between the platelet count in the control group and subgroups of HBV-related liver disease showed interesting findings. Cirrhotic patients had a mean platelet count that is in the thrombocytopaenic range (105 ± 23). This is the expected abnormality of platelet count in cirrhosis. The mechanisms responsible for the thrombocytopaenia of cirrhosis include splenic sequestration of platelets in portal hypertension [23, 24], suppression of platelet production in the bone marrow [25, 26] and decreased activity of the hematopoietic growth factor, thrombopoietin [27], which is produced primarily in the liver. There is also increased destruction of platelets by immunological mechanisms that result from increased levels of platelet-associated immunoglobulins (PAIgG). Increased levels of PAIgG have been reported in 55-88% of patients with chronic liver disease [28, 29]. Furthermore antiviral therapy with interferon alpha induces thrombocytopaenia, necessitating dose reduction in some instances [30].

Chronic hepatitis caused by HBV and hepatitis C virus HCV) has been documented as a possible cause of thrombocytopenia, even in the absence of cirrhosis [31]. This was however not demonstrated in this study as there was no statistically significant difference between the mean platelet counts in patients with chronic hepatitis and control subjects. There are 2 possible explanations for this. First, the number of patients with chronic hepatitis in this study was relatively small (8.4%), and secondly, chronic hepatitis is a very heterogeneous group determined by the extent of necroinflammation and fibrosis in the liver. It is possible that most of the chronic hepatitis patients in this study were in the early stages, with little or no necroinflammation and fibrosis. The average Child-Pugh’s score of 5.6 ± 0.7 in this group lends credence to the explanation.

The mean platelet count in patients with HCC was 303 ± 160. This was significantly higher than the mean platelet count in the control subjects, asymptomatic infection, chronic hepatitis or cirrhosis. Three patients with HCC actually had thrombocytosis (6.1%). Hepatocellular carcinoma develops in a cirrhotic liver in 80% of cases and this preneoplastic state is thought to be the strongest predisposing factor [32]. However the incidence of liver decompensation from hepatocellular failure in patients with HCC is variable. When cirrhosis and liver decompensation are the dominant features, signs and symptoms of liver failure and portal hypertension take a leading position whereas in their absence features coinciding with the “toxic syndrome” prevail [33-35]. This syndrome consists of asthenia, malaise, fever, anorexia and weight loss from malignant cachezia. Therefore, depending on the pattern of presentation of the underlying cirrhosis, thrombocytopaenia may not be a dominant feature. If anything, thrombocytosis may predominate from other mechanisms.

Thrombocytosis is common in many malignant diseases. The prevalence of thrombocytosis in patients with primary lung cancer was 53% in one study [36]. An increased preoperative platelet count has been correlated with prognosis in several malignancies including bronchial [4], gastric [5], gynaecological [6, 7], and colorectal [8] cancers. Thrombocytosis has also been shown to be an independent prognostic indicator of survival in patients undergoing resections for squamous cell carcinoma of the oesophagus [9] and adenocarcinoma of the pancreas [10, 11].

Thrombocytosis has been found in children with hepatoblastoma [37-39]. A prevalence of 6.1% for thrombocytosis in patients with HCC in this study is an important observation. Though thrombopoietin levels were not done, the thrombocytosis may be a paraneoplastic manifestation of HCC.

The explanation for increased platelet count in patients with malignancies may lie in the link between platelets and angiogenesis [40-42]. Various types of tumor can activate platelets in vitro by virtue of direct contact, release of ADP, production of thromboxane A_2_ or cancer procoagulant, generation of thrombin, or activation of the tumor-associated proteinases [41]. In the presence of vascular endothelial growth factor (VEGF), which is a platelet-derived cytokine, endothelial cells promote platelet aggregation [43]. Adhesion and aggregation of activated platelets are accompanied by the release of many potential angiogenesis regulators such as VEGF-A [44], VEGF-C [45] and platelet-derived endothelial cell growth factor (PD-ECGF) [46]. These observations suggest that platelets may play an active and causative role in tumor angiogenesis [42].

However, a lot remains to be unraveled in the subject because platelets are also known to elaborate angiogenesis inhibitors such as platelet factor-4 (PF4) [47] and thrombospondin-1 [48]. The latter is also known to stimulate angiogenesis in very high concentrations [48].

Platelet activating factor (PAF)-receptor antagonists have exhibited promising results in vitro and in vivo as anti-angiogenic molecules in cancer cells and tumors [49]. However, the great complexity of the haemostatic system and the multitude of pro- and anti-angiogenic activities it encodes (even within individual constituent proteins) suggest that for each therapeutic action, a proper clinical context should be precisely defined and validated.

In conclusion, we have demonstrated that disorders of platelet count are common in liver disease caused by HBV infection. Thrombocytopenia tends to predominate in patients with cirrhosis whereas higher counts, including thrombocytosis are more common in patients with HCC. Apart from the potential utility of these findings in follow up and prognostication, there are prospects of exploiting them for therapeutic gain.
